# Integrating proteomic data with metabolic modeling provides insight into key pathways of *Bordetella pertussis* biofilms

**DOI:** 10.3389/fmicb.2023.1169870

**Published:** 2023-08-03

**Authors:** Hiroki Suyama, Laurence Don Wai Luu, Ling Zhong, Mark J. Raftery, Ruiting Lan

**Affiliations:** ^1^School of Biotechnology and Biomolecular Sciences, University of New South Wales, Sydney, NSW, Australia; ^2^Bioanalytical Mass Spectrometry Facility, University of New South Wales, Sydney, NSW, Australia

**Keywords:** *Bordetella pertussis*, proteomics, metabolic model, infectious disease, label free quantification (LFQ), mass spectrometry, whooping cough

## Abstract

Pertussis, commonly known as whooping cough is a severe respiratory disease caused by the bacterium, *Bordetella pertussis*. Despite widespread vaccination, pertussis resurgence has been observed globally. The development of the current acellular vaccine (ACV) has been based on planktonic studies. However, recent studies have shown that *B. pertussis* readily forms biofilms. A better understanding of *B. pertussis* biofilms is important for developing novel vaccines that can target all aspects of *B. pertussis* infection. This study compared the proteomic expression of biofilm and planktonic *B. pertussis* cells to identify key changes between the conditions. Major differences were identified in virulence factors including an upregulation of toxins (adenylate cyclase toxin and dermonecrotic toxin) and downregulation of pertactin and type III secretion system proteins in biofilm cells. To further dissect metabolic pathways that are altered during the biofilm lifestyle, the proteomic data was then incorporated into a genome scale metabolic model using the Integrative Metabolic Analysis Tool (iMAT). The generated models predicted that planktonic cells utilised the glyoxylate shunt while biofilm cells completed the full tricarboxylic acid cycle. Differences in processing aspartate, arginine and alanine were identified as well as unique export of valine out of biofilm cells which may have a role in inter-bacterial communication and regulation. Finally, increased polyhydroxybutyrate accumulation and superoxide dismutase activity in biofilm cells may contribute to increased persistence during infection. Taken together, this study modeled major proteomic and metabolic changes that occur in biofilm cells which helps lay the groundwork for further understanding *B. pertussis* pathogenesis.

## Introduction

Whooping cough is a re-emerging severe respiratory disease caused by *Bordetella pertussis*. Following the change from the whole cell vaccine (WCV) to the acellular vaccine (ACV) in many developed countries, there has been an increase in the incidence of whooping cough (de Melker et al., 1997; Güriş et al., 1999; Galanis et al., 2006; Campbell et al., 2015). Although most likely multifaceted, waning immunity of the ACV and vaccine driven selection of non-ACV genotypes or strains not expressing one of the ACV antigens have been previously reported as major factors contributing to the re-emergence of pertussis (Octavia et al., 2011; Mooi et al., 2014). It is evident that an improved ACV is needed to control the infections.

Recent studies have shown that *B. pertussis* readily forms biofilms *in vivo* (Soane et al., 2000; Mishra et al., 2005; Paddock et al., 2008; Conover et al., 2010, 2011; Serra et al., 2011; Cattelan et al., 2017). Development of the vaccine has been based on planktonic studies and may not be entirely representative of the infection cycle. Although proteomic comparisons have been performed between biofilm and planktonic *B. pertussis* cells (Serra et al., 2008; de Gouw et al., 2014; Arnal et al., 2015; Dorji et al., 2016; Carriquiriborde et al., 2021), little is known about the metabolic reactions that are altered while in the biofilm state. In response to changes in environment, the most widely studied regulator of gene expression in *B. pertussis* is the *Bordetella* virulence gene (Bvg) system (Moon et al., 2017). The Bvg system controls the expression of most of the virulence factors in *B. pertussis* but has also been implicated in the regulation of metabolism (Belcher et al., 2020). The Bvg system exists in 3 states, Bvg^+^, Bvg^−^ and Bvg^i^ where the expression of virulence genes is active, inactive or intermediately expressed, respectively. There are conflicting studies surrounding the role of the Bvg system in the biofilm process, but studies have linked *Bordetella* biofilm with the Bvg^i^ phase (Irie et al., 2004; Serra et al., 2008; Nicholson et al., 2012; Sisti et al., 2013; de Gouw et al., 2014; Arnal et al., 2015). Further studies identifying key changes in protein expression and metabolism in biofilm cells can provide an insight into the capabilities of the pathogen and help with understanding the role of biofilms in *B. pertussis* pathogenesis.

Genome scale metabolic models (GSMM) have emerged as a powerful tool in understanding the metabolic capabilities of an organism. The creation of a GSMM begins as a draft network based on annotated enzyme data and the genome. This yields a model with a network of reactions and metabolites within a mathematical matrix. The movement of metabolites through the network is defined as flux, i.e., the rate at which the metabolites are consumed or produced. Based on stoichiometric and thermodynamic constraints, permissible minimum and maximum flux values for each reaction can be calculated (Orth et al., 2010).

GSMMs have been successfully utilised in several pathogens such as *Salmonella enterica* serovar Typhimurium (Fong et al., 2013), *Listeria monocytogenes* (Lobel et al., 2012; Metz et al., 2018), *Staphylococcus aureus* (Lee et al., 2009) and *Mycobacterium tuberculosis* (Rienksma et al., 2018) to predict important pathways for virulence and growth. Currently, there have been over 6,000 organisms that have been metabolically reconstructed either manually or automatically (Gu et al., 2019). There have been two GSMMs extensively curated for *B. pertussis* (Branco Dos Santos et al., 2017; Fyson et al., 2017). These models showed the metabolic versatility of the organism by identifying minimal nutrient requirements. Additionally, both models were utilised to validate key pathways essential for infection (Gonyar et al., 2019). The major drawback of these models is the assumption that all protein products are simultaneously expressed. Many physical and chemical constraints make this assumption unlikely to be true. Additionally, the different conditions in which the organism is grown strongly affects the metabolic processes (Åkesson et al., 2004).

To predict metabolic reactions reflecting a specific phenotype, such as biofilms, a context specific model should be created. This can be done through the integration of ‘omics’ expression data into the GSMM to enrich pathways reflective of the context (i.e., biofilm). The Integrative Metabolic Analysis Tool (iMAT) (Zur et al., 2010) is a method that has been developed to incorporate expression data within a metabolic model. The iMAT algorithm uses a mixed integer linear programming (MILP) problem to enrich pathways based on the expression data while maintaining a steady flux distribution and the stoichiometric and thermodynamic constraints. A major advantage of the iMAT method is the incorporation of the expression data as accumulated cues for a base model. By applying the expression data as influential factors rather than forcing flux through the associated reactions, the method accounts for experimental limitations such as missing proteins or errors in expression measurements (Shlomi et al., 2008). The iMAT model not only creates a phenotype specific model but post-transcriptional regulation can be predicted through this model as the surrounding fluxes would indicate the relative activity of an enzyme in a pathway (Zur et al., 2010). Potential changes in metabolic reactions between phenotypes can be identified by comparing the predicted flux distributions between two context specific models (Shlomi et al., 2008; Stempler et al., 2014).

In this study, proteomic expression data was used to compare biofilm and planktonic *B. pertussis* cells from a representative current circulating strain. Furthermore, the protein expression data was incorporated into a GSMM to create context specific iMAT models to elucidate key metabolic changes that may allow biofilm cells to persist in the host.

## Methods

### Bacterial strains and biofilm growth

A clinical *Bordetella pertussis* strain, L1423 isolated from the 2008–2012 Australian epidemic with genotype *ptxP3/ptxA1/fim3A/prn2* and expressing pertactin, was used as a representative of the predominant cluster I strains (Safarchi et al., 2016). The genome has been previously sequenced, and the strain has been used in two separate infection studies in mice (Safarchi et al., 2015, 2016). *B. pertussis* cells were grown using a previously established method and proteins were extracted (Luu et al., 2018). Briefly, the *B. pertussis* strain was grown on Bordet-Gengou agar (BG, BD Scientific) for 3–5 days at 37°C. A loopful of pure Bvg^+^ colonies were suspended in 20 mL Thalen-IJssel (THIJS) media (Thalen et al., 1999) supplemented with 1% heptakis [(2,6-O-dimethyl) β-cyclodextrin] and 1% THIJS supplement in 50 mL TPP TubeSpin Bioreactor tubes (Merck). Cells were grown for 24 h shaking at 180 rpm at 37°C. For planktonic growth, the OD_600_ of the starter culture was adjusted to 0.05/mL in 20 mL THIJS and incubated under the same conditions as above for 12 h [reaching log phase for L1423 (Luu et al., 2017)]. For biofilms, the OD_600_ was adjusted to 0.1/mL in THIJS media and 1 mL of this adjusted culture was seeded into each well of a 24 well polystyrene plate (Dorji et al., 2016; Hoffman et al., 2017). The 24 well plate was incubated statically for 5 h at 37°C for attachment of cells before the media was refreshed to remove non-adherent cells (Serra et al., 2008; de Gouw et al., 2014). After 96 h of incubation under agitation (60 rpm), the wells were washed with PBS and then the plate was water bath sonicated at 37 kHz for 2 min to detach cells (Bjerkan et al., 2009; Noorian et al., 2017). The planktonic and biofilm cells were then probe sonicated and proteins extracted as described by Luu et al. (2018). Six biological replicates per condition were performed.

### Confocal laser scanning microscopy analysis

To confirm biofilm maturity, confocal laser scanning microscopy (CLSM) was used. This method was adapted from Cattelan et al. (2018). The *B. pertussis* cells were grown on a glass coverslip angled at 45° in the same method as described above. The biofilm was imaged at 24, 48, 72, and 96 h. The biofilm was fixed with 4% paraformaldehyde and stained with SYTO 9 (Thermo Fisher Scientific) fluorescent dye. The coverslips were imaged on the FluoView FV1200 inverted confocal microscope (Olympus Life Sciences) at the UNSW Katharina Gaus Light Microscopy Facility (KG-LMF). Three biological replicates were performed per time point and 3 field of views per replicate were randomly selected for z-stack 3D imaging. The biomass, average thickness and maximum thickness were calculated using the COMSTAT2 (v 2.1) ImageJ (v 2.8.0) plugin (Heydorn et al., 2000).

### Protein preparation and LC–MS/MS

Ten micrograms of protein extract from biofilm and planktonic cells were reduced with dithiothreitol, alkylated with iodoacetamide and then digested with trypsin as described in Luu et al. (2017). The peptides were analyzed on the LTQ-Orbitrap Velos mass spectrometer (Thermo Fisher Scientific) at the UNSW Bioanalytical Mass Spectrometry Facility (BMSF) with the settings described in Luu et al. (2017). The output spectra were matched against a custom *B. pertussis* database (consists of Tohama I, CS, B1917 and B1920 protein sequences) on the MaxQuant (v2.0.3.1) proteomics software with the following parameters: digestion mode – specific, enzyme – Trypsin/P, variable modification – oxidation (M), fixed modification – carbamidomethyl (C), max missed cleavages – 1, Label free quantification – LFQ, Protein identification false discovery rate – 0.01 and min peptides per protein – 2. All other parameters were set as the recommended default values. Student’s *t*-test was calculated and a false discovery rate (FDR) *q*-value multiple test correction was performed using the Storey-Tibshirani method on R (v4.1.1) (Storey and Tibshirani, 2003). Proteins were considered upregulated if the fold change (FC) was >1.2, *q* < 0.05 and downregulated if FC < 0.8, *q* < 0.05 based on previous studies (Luu et al., 2018). Functional categories were assigned to proteins based on Bart et al. (2014). The mass spectrometry proteomics data have been deposited to the ProteomeXchange Consortium via the PRIDE (Perez-Riverol et al., 2022) partner repository with the dataset identifier PXD033664 and DOI 10.6019/PXD033664.

### Integrative Metabolic Analysis Tool (iMAT) model generation

The proteomic expression data was used to generate context specific metabolic models for the planktonic and biofilm cells. The iMAT (Zur et al., 2010) method, available in the COBRA Toolbox (v3.0) (Heirendt et al., 2019), was used. Processing was done in MATLAB (R2020a) using the IBM CPLEX optimiser (v12.10.0). The iMAT algorithm extracts a simplified model based on the trade-off between high expression and low expression reactions. The protein expression data were not used as absolute values but used as cues for the likelihood that its associated reaction carries metabolic flux (Shlomi et al., 2008; Zur et al., 2010). The comprehensive, manually curated *B. pertussis* metabolic model (iBP1870) generated by Branco Dos Santos et al. (2017) was used as the base model. Subsystems from the *Escherichia coli* genome scale metabolic model (iAF1260) were assigned to each of the reactions in the *B. pertussis* model (Feist et al., 2007). The *E. coli* (iAF1260) model was used as a template for the original *B. pertussis* model (iBP1870) and therefore, most of the subsystems are transferrable. The original *B. pertussis* iBP1870 model had the tricarboxylic acid (TCA) cycle partially dysfunctional (no flux from oxaloacetate to ⍺-ketoglutarate), as this model was based on previous studies which stated *B. pertussis* has an incomplete TCA cycle (Thalen et al., 1999). However, recent studies have shown that the TCA cycle is fully functional and therefore the disabled pathways were activated before generating the iMAT models (Izac et al., 2015). After refining the *B. pertussis* model, the biofilm and planktonic proteins were assigned to the gene-protein-reaction (GPR) associations listed in the *B. pertussis* model (iBP1870).

**Figure 1:**
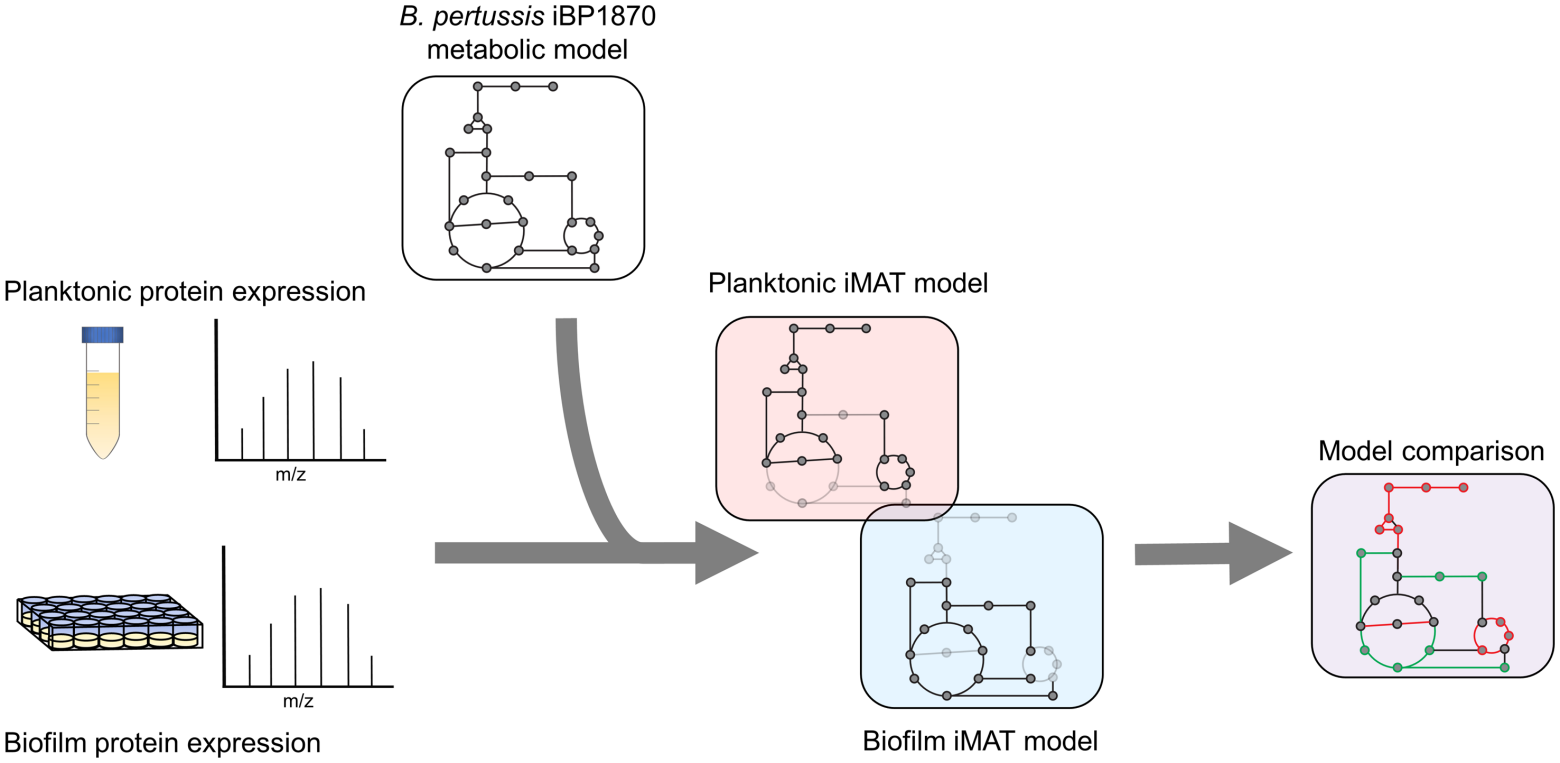
Experimental design for iMAT model generation. Proteins were extracted from planktonic and biofilm *B. pertussis* cells. The expression values were then used to generate context specific metabolic models based on the extensively curated iBP1870 *B. pertussis* model (322). Flux balance analysis was run on the models and the altered reactions compared between the planktonic and biofilm models.

Label free quantification (LFQ) intensity values designated by the MaxQuant software were used to estimate relative protein abundance. To increase confidence in the imported data, only proteins identified in all six biological replicates per condition were incorporated. Proteins were designated as uniquely identified if found in all 6 biological replicates in one condition and none in the other. The expression data was defined as highly (+1), lowly (−1) or moderately (0) expressed based on the threshold of mean expression ±0.5 x STD of the proteins included in the model (Zur et al., 2010). After generating the planktonic and biofilm context specific iMAT models, the reactions in the biofilm and planktonic models were compared (Figure 1).

Additional models were generated with proteomic expression data from a separate study. To our knowledge, there are 5 studies that have compared protein expression of *B. pertussis* biofilm cells with their planktonic counterpart (Serra et al., 2008; de Gouw et al., 2014; Arnal et al., 2015; Dorji et al., 2016; Carriquiriborde et al., 2021). Of these, only one study by de Gouw et al. (2014) has publicly available global proteomic expression data of planktonic and biofilm cells and thus was used for the production of iMAT models. The proteomic study by de Gouw et al. (2014) compared *B. pertussis* biofilm and planktonic (mid-log and stationery) cytosolic and membrane proteins. The averaged expression values of the cytosolic and membrane fractionated proteins were combined, and mid-log planktonic expression values were used to generate the models. All models have been deposited to the BioModels database (Malik-Sheriff et al., 2019) in SBML L3V1 format under the model identifier MODEL2205270001. Differences between the growth conditions are listed in Supplementary Table S1.

### Flux analysis

A flux variability analysis (FVA) (Mahadevan and Schilling, 2003) using the COBRA Toolbox (v3.0) (Heirendt et al., 2019) was performed on the models which provides minimum and maximum permissible flux bounds for each reaction. Flux is recorded as a millimoles of metabolite per gram of dry cell weight per hour (mmol · g_DCW_^−1^ · h^−1^). To compare the similarity in flux bounds between the biofilm and planktonic models, the Jaccard index function within the COBRA Toolbox (v3.0) (Heirendt et al., 2019) was used. This process assigns a similarity index (1 = most similar) by comparing the minimum and maximum flux bounds for the common reactions between the two models for each individual reaction. Many of the reactions have a forward and/or reverse directionality. This is represented in the model as a positive (forward) and negative (reverse) flux value. The FVA values are helpful in determining the predicted directionality of the reaction in the model as flux bounds would often be limited to negative or positive values.

Each model contains a complete set of reactions, however, not all reactions are essential to the model. If a reaction that is essential to the model is missing, the FVA function in the COBRA Toolbox (v3.0) (Heirendt et al., 2019) states that the model is infeasible. To identify the most important reactions in each model, each reaction was individually removed in turn and an FVA calculation was attempted to determine whether the model would still be feasible without the reaction.

A flux balance analysis (FBA) was performed using COBRA Toolbox (v3.0) (Heirendt et al., 2019) to identify changes in flux in reactions shared by the planktonic and biofilm models. The FBA provides a single value for each reaction based on a predefined metabolic goal so that the models may be compared. The FBA method has been extensively developed to predict metabolic fluxes within metabolic models (Orth et al., 2010). Typically, the objective function (*c*) of the linear optimisation equation is set as an artificial biomass reaction as the metabolic goal of the organism. As generating biomass may not be the metabolic objective of biofilm cells, an alternate method for defining the objective function was used in this study. The objective function was defined based on proteomic expression data as described in Montezano et al. (2015). For all proteins that were identified with GPR associations in the model, the intensities were normalized by the maximum intensity value for each condition (planktonic and biofilm) and these values were input as *c*. This leads the FBA to push flux toward the reactions which have higher protein expression and considerably shrinks the solution space to increase prediction accuracy (Montezano et al., 2015).

## Results

### Key proteomic changes identified between biofilm and planktonic cells

**Figure 2:**
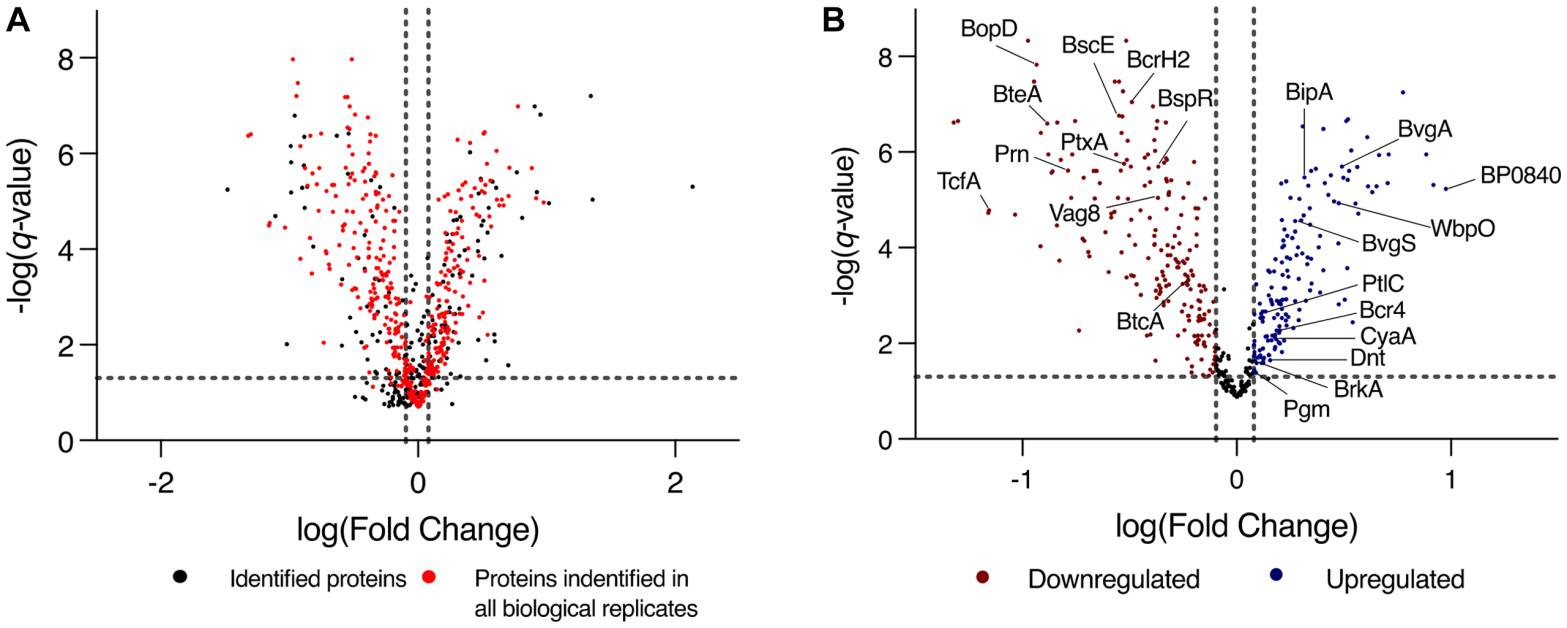
Volcano plot of total protein expression changes between biofilm and planktonic cells. **(A)** Expression profile of all proteins identified through the LC–MS/MS analysis of planktonic and biofilm *B. pertussis* cells. Proteins are plotted on a volcano plot displaying the -log(*q*-value) on the *y*-axis and log[fold change (biofilm/planktonic)] on the *x*-axis. The dashed vertical gray lines mark a fold change of 0.8 and 1.2 and the horizontal line marks the threshold of *q*-value = 0.05. Highlighted in red are the proteins that were identified in all 6 biological replicates that were incorporated into the iMAT models. **(B)** Expression profile of the subset of proteins incorporated into the iMAT model. Red markers are proteins that were designated significantly differentially downregulated, and the blue markers are proteins that were significantly upregulated in biofilm cells. The dashed vertical gray lines mark a fold change of 0.8 and 1.2 and the horizontal line marks the threshold of *q*-value = 0.05. Proteins of interest are labeled.

To identify changes that occur in biofilm conditions, label free quantification mass spectrometry (LFQ-MS) was performed on biofilm and planktonic cells. Analysis was performed on L1423, a clinical isolate representative of the current circulating *B. pertussis* strains. Confocal microscopy confirmed biofilm formation and mature structure at 96 h (Supplementary Figure S1). Furthermore, there was a polysaccharide biosynthesis protein, WbpO (FC = 2.98, *q* = 1.17E-5), a phosphoglucomutase enzyme, Pgm (FC = 1.23, *q* = 0.041) and an outer membrane porin protein, BP0840 (FC = 9.45, *q* = 1.05E-5) that were seen to be upregulated in biofilm cells (Figure 2B). These proteins have been previously linked with *B. pertussis* biofilm reinforcing the biofilm phenotype achieved in this study (Serra et al., 2008). There were 948 proteins identified in total (Supplementary Table S2), of which, 571 were proteins identified in all 6 biological replicates (Figure 2A). There were 478 proteins with significantly differential expression (*q* < 0.05) between the two conditions. In biofilm cells, there were 242 proteins downregulated and 236 proteins upregulated (Figure 2B).

There were many proteins related to virulence with altered expression identified in this study (Table 1). BipA, an outer membrane protein associated with biofilm formation was upregulated (de Gouw et al., 2014). Adenylate cyclase toxin (CyaA) and dermonecrotic toxin (Dnt) were also upregulated in biofilm (Figure 2B). There were 10 proteins from the type III secretion system (T3SS) that were downregulated in biofilm cells or uniquely identified in planktonic cells. One protein (Bcr4) involved in the T3SS was upregulated in biofilm cells (Goto et al., 2021). Tracheal colonisation factor (TcfA) and virulence associated gene 8 (Vag8) were downregulated in biofilm cells while *Bordetella* resistance to killing (BrkA) protein was upregulated (Figure 2B). Of the ACV antigens, there was no change in expression for filamentous haemagglutinin (FhaB) but the filamentous haemagglutinin outer membrane transporter protein (FhaC) was uniquely identified in biofilm cells. The membrane bound pertussis toxin subunit 4 (PtxD) was also uniquely identified in biofilm cells, however, pertussis toxin subunit 1 (PtxA) was downregulated in biofilms. Furthermore, fimbriae protein (Fim2) was downregulated in biofilm. Finally, pertactin (Prn) was strongly downregulated in biofilm (FC = 0.16, *q* = 2.42E-6). These changes demonstrate a strongly altered virulence profile in *B. pertussis* biofilm cells compared to planktonic cells.

**Table 1:** Virulence proteins differentially expressed or uniquely identified between planktonic and biofilm cells.

Locus	Gene	Product	Fold change (Biofilm/Planktonic)	*t*-test (*p* ≤ 0.05)	*q*-value (*q* ≤ 0.05)
BP0499	*btcA*	Type III secretion chaperone	0.594	0.002	0.0005
BP0500	*bteA*	Type III secretion toxin, effector	0.131	9.02E-08	2.52E-07
BP0760	*cyaA*	Bifunctional hemolysin-adenylate cyclase	1.530	0.042	0.008
BP1054	*prn*	Pertactin autotransporter	0.163	2.19E-06	2.42E-06
BP1112	*bipA*	Outer membrane ligand binding protein	2.073	3.65E-06	3.4E-06
BP1119	*fim2*	Serotype 2 fimbrial subunit	0.590	0.002	0.0006
BP1201	*tcfA*	Tracheal colonisation factor	0.070	2.94E-05	1.64E-05
BP1251	*–*	Putative toxin	Planktonic unique		
BP1877	*bvgS*	Virulence sensor protein BvgS	1.876	5.63E-05	2.77E-05
BP1878	*bvgA*	Virulence factors transcription regulator BvgA	3.101	1.69E-06	2.02E-06
BP1884	*fhaC*	Filamentous hemagglutinin transporter protein FhaC	Biofilm unique		
BP2233	*bspR*	Type III secretion chaperone	0.430	1.66E-06	2.02E-06
BP2236	*bscW*	Type III secretion chaperone	Planktonic unique		
BP2250	*bcr4*	Type III secretion protein	1.533	0.027	0.005
BP2251	*bcrH2*	Type III secretion chaperone	0.324	1.38E-08	8.98E-08
BP2253	*bopD*	Type III secretion system outer protein D	0.116	7.61E-10	1.49E-08
BP2255	*btc22*	Type III secretion protein chaperone	Planktonic unique		
BP2256	*bsp22*	Type III secretion tip protein	Planktonic unique		
BP2257	*bopN*	Type III secretion outer protein N, effector	Planktonic unique		
BP2263	*bscE*	Type III secretion protein	0.282	3.24E-08	1.73E-07
BP2315	*vag8*	Autotransporter	0.429	1.39E-05	9.03E-06
BP3439	*dnt*	Dermonecrotic toxin	1.429	0.135	0.022
BP3494	*brkA*	BrkA autotransporter	1.218	0.139	0.023
BP3654	*cyaY*	Iron–sulfur cluster assembly protein CyaY	0.618	0.188	0.030
BP3783	*ptxA*	Pertussis toxin subunit 1	0.299	1.37E-06	1.75E-06
BP3785	*ptxD*	Pertussis toxin subunit 4	Biofilm unique		

**Figure 3:**
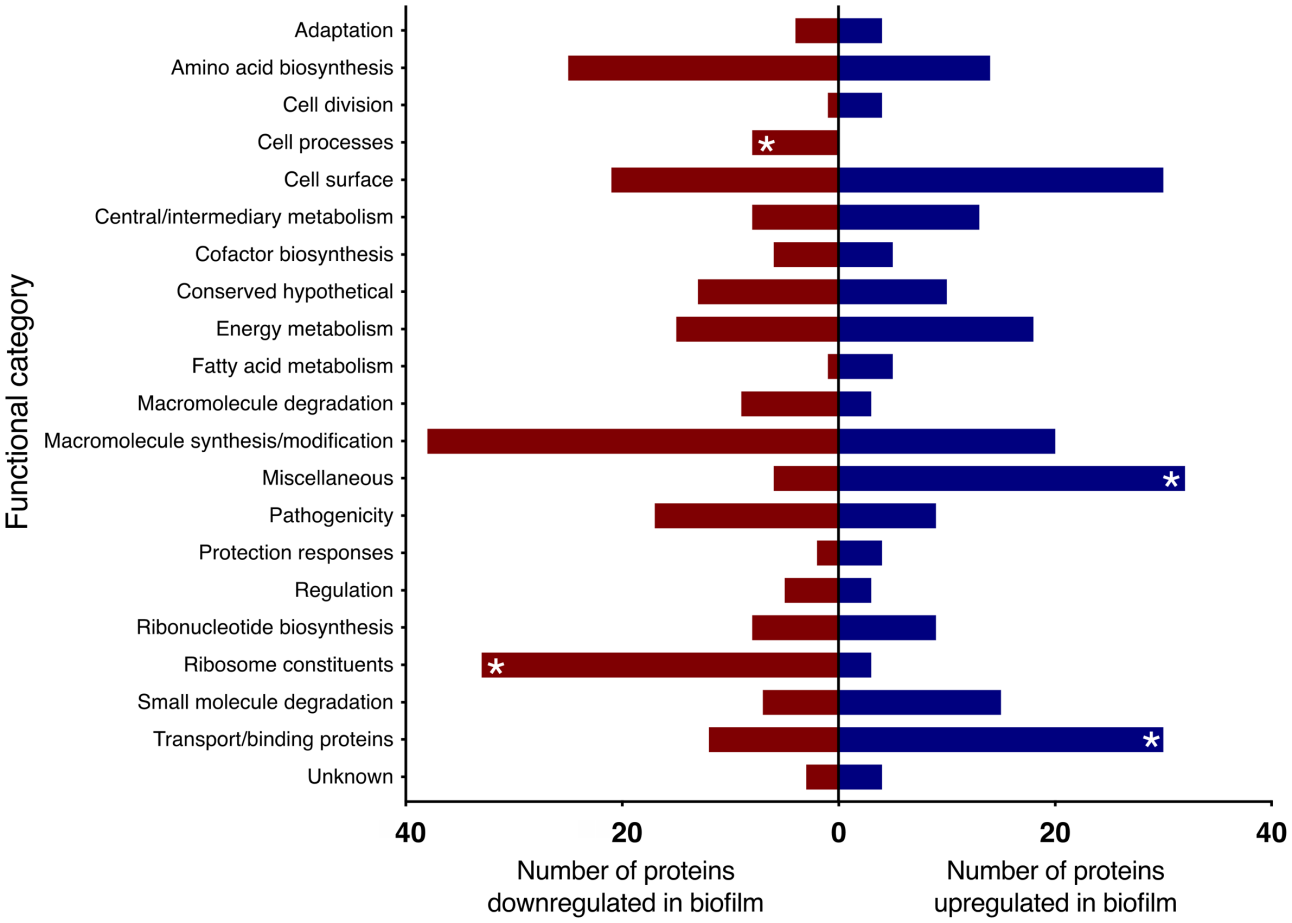
Proteins up and downregulated in *B. pertussis* biofilm cells compared to planktonic cells identified using LC–MS/MS. Proteins significantly up and downregulated in biofilm cells were categorised in functional categories based on Bart et al. (2014). Red and blue bars represent the total number of proteins within the functional category significantly up or downregulated, respectively. Asterisk (*) denotes functional categories significantly up or downregulated based on Fisher’s exact test with Benjamini-Hochberg multiple test correction (adjusted *p* < 0.05).

When the proteins were grouped into functional categories based on Bart et al. (2014), there was a significant increase in proteins in the categories of transport/binding and miscellaneous proteins under biofilm conditions. There was also a downregulation of the functional groups: ribosome constituents and cell processes (Figure 3).

### iMAT model generation

**Figure 4:**
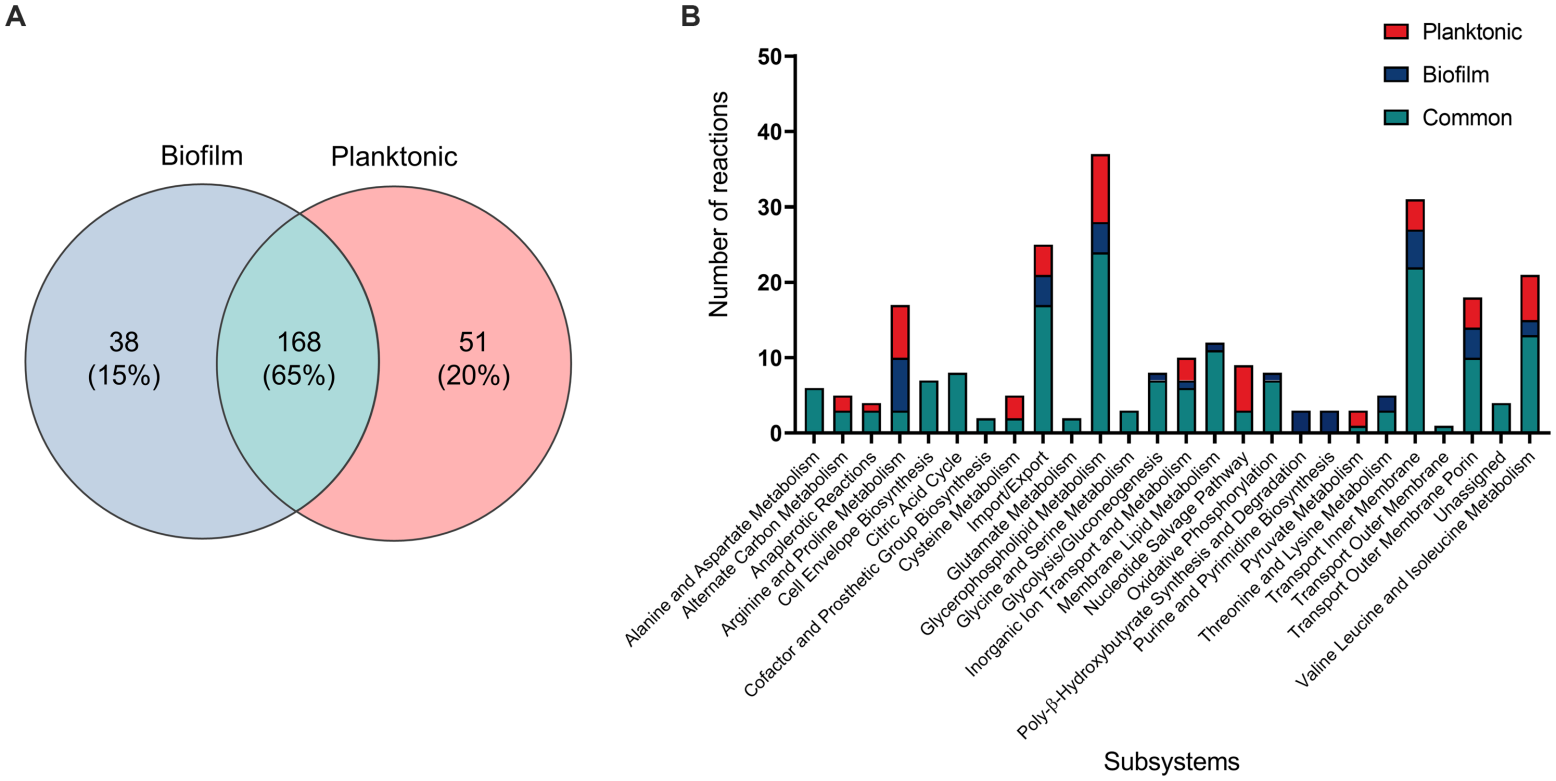
Comparison of reactions of *B. pertussis* planktonic and biofilm iMAT models. **(A)** Venn diagram highlighting common and unique reactions between planktonic and biofilm iMAT models generated by incorporating proteomic expression data. **(B)** Planktonic and biofilm model reactions grouped into subsystems based on the *Escherichia coli* iAF1260 metabolic model. Unique reactions identified in one model but not the other are highlighted in different colors on the graph.

**Figure 5:**
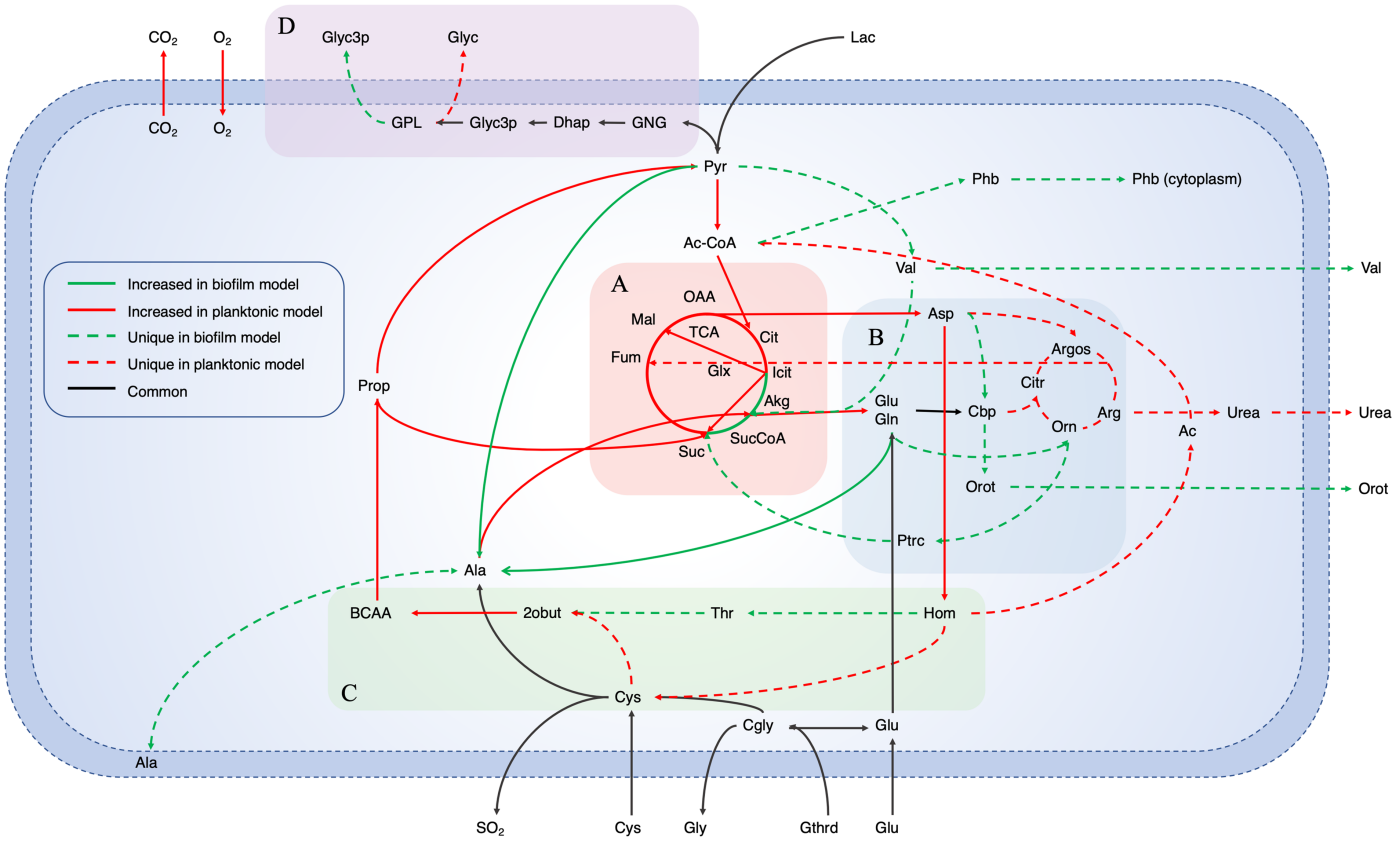
Summarized pathways with major changes between planktonic and biofilm iMAT models. Major changed core metabolic reactions from a comparison of iMAT models generated from biofilm and planktonic *B. pertussis* protein expression data. The model includes the reactions that were unique in either model as well as reactions with altered flux. Red lines indicate reactions that had decreased flux in biofilm cells while green lines indicate reactions with increased flux. The dashed lines indicate unique reactions to each model while the black lines are common reaction with the same flux. Highlighted sections are **(A)** the Tricarboxylic acid cycle, **(B)** Arginine metabolism, **(C)** Aspartate metabolism and **(D)** Gluconeogenesis and glycerophospholipid metabolism. Pathways for **(A–C)** are represented in more detail in Figures 6–8, respectively. Ac, Acetate; Ac-CoA, Acetyl-CoA; Akg, α-ketoglutarate; Ala, Alanine; Arg, Arginine; Argos, Argininosuccinate; Asp, Aspartate; BCAA, Branched chain amino acid degradation; Cbp, Carbamoyl phosphate; Cgly, Cysteinylglycine; Cit, Citrate; Citr, Citruline; CO2, Carbon dioxide; Cys, Cysteine; Dhap, Dihydroxyacetone phosphate; Fum, Fumarate; Gln, Glutamine; Glu, Glutamate; Glx, Glyoxylate; Gly, Glycine; Glyc3p, Glycerol 3-phosphate; Glyc, Glycerol; GNG, Gluconeogenesis; GPL, Glycerophospholipid metabolism; Gthrd, Glutathione; Hom, Homoserine; Icit, Isocitrate; Lac, Lactate; Mal, Malate; O2, Oxygen; OAA, Oxaloacetate; Orn, Ornithine; Orot, Orotate; Phb, Polyhydroxybutyrate; Prop, Propanoate metabolism; Ptrc, Putrescine; Pyr, Pyruvate; SO2, Sulfur dioxide; Suc, Succinate; SucCoA, Succinyl-CoA; TCA, Tricarboxcylic acid cycle; Thr, Threonine; Val, Valine; 2obut, 2-oxobutanoate.

**Figure 6:**
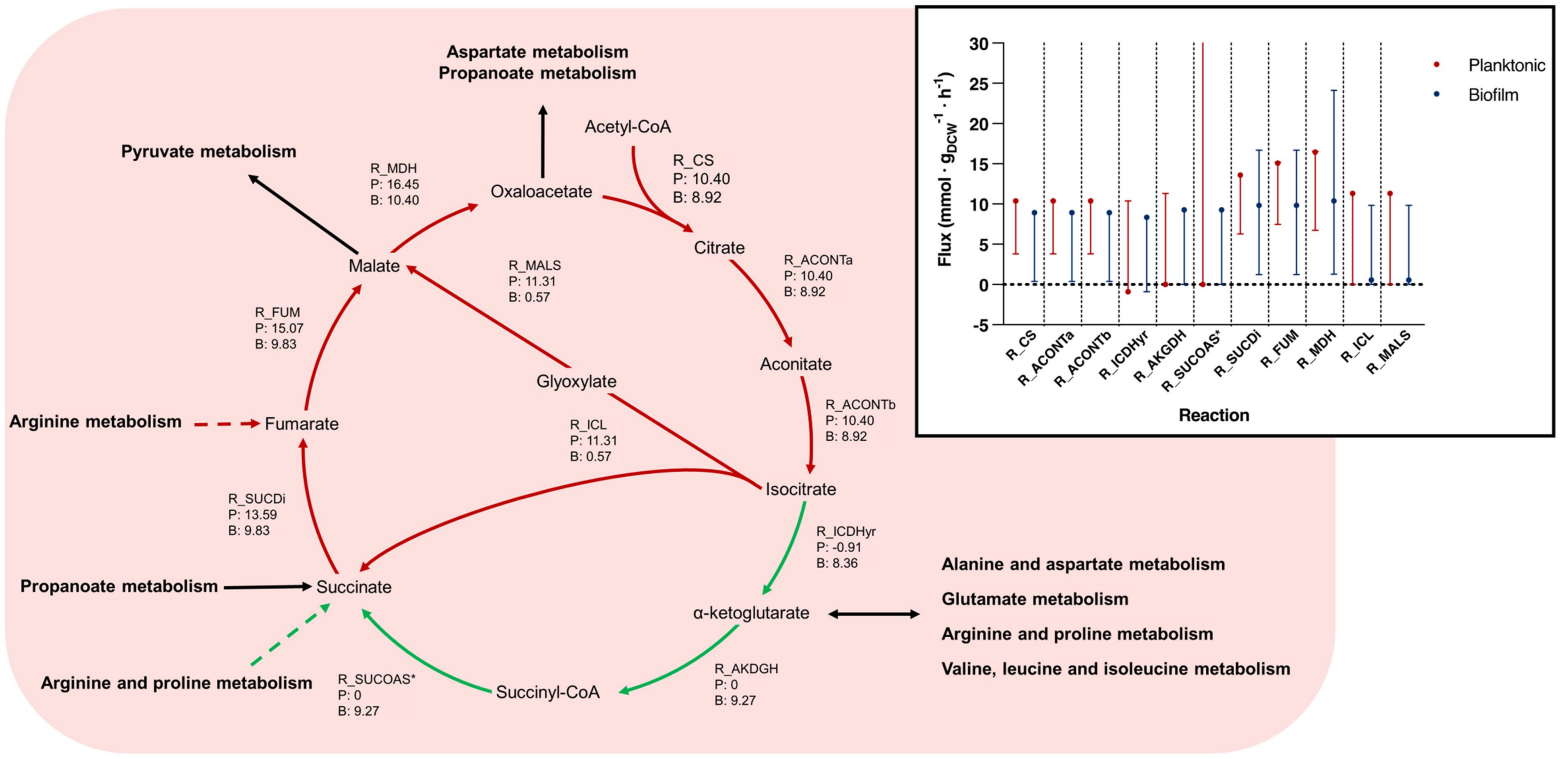
Tricarboxylic acid cycle pathways and flux bounds between planktonic and biofilm iMAT models. This figure relates to Figure 5A. Reactions within the tricarboxylic acid (TCA) cycle between planktonic and biofilm *B. pertussis* iMAT metabolic models generated from proteomic expression data. Reaction names and flux values from the flux balance analysis (FBA) are given for each reaction within the TCA. Each arrow is indicates a reaction and flux values are labeled as (P) representing planktonic model flux and (B) representing biofilm model flux. All flux values are mmol · g_DCW_^−1^ · h^−1^. Green arrows represent reactions that are upregulated in biofilm cells while red arrows are downregulated reactions. Green dashed arrows are unique reactions to the biofilm model while red dashed arrows are reactions unique to the planktonic model. Black arrows are common reactions. General metabolic pathways are in bold. Inset Range for flux variance analysis and FBA values for the tricarboxylic acid cycle between planktonic and biofilm cells. Flux ranges (min to max) are represented as lines and the FBA value is annotated with a point. Planktonic values are indicated in red and biofilm values in blue. *R_SUCOAS is a reaction that flows from succinate to succinyl-CoA. The reaction runs in reverse in the typical TCA cycle and so these values are negative. For simplicity, these reactions have been annotated as absolute values in the figure. Furthermore, the flux bounds for the planktonic models are at the max value and the max has been omitted from the graph. R_CS, type II citrate synthase; R_ACONTa, citrate hydrolase; R_ACONTb, aconitate hydratase; R_ICDHyr, isocitrate dehydrogenase; R_AKGDH, α-ketoglutarate dehydrogenase; R_SUCOAS, succinyl-CoA synthetase; R_SUCDi, succinate dehydrogenase; R_FUM, fumarate hydratase; R_MDH, malate dehydrogenase; R_ICL, isocitrate lyase; R_MALS, malate synthase.

**Figure 7:**
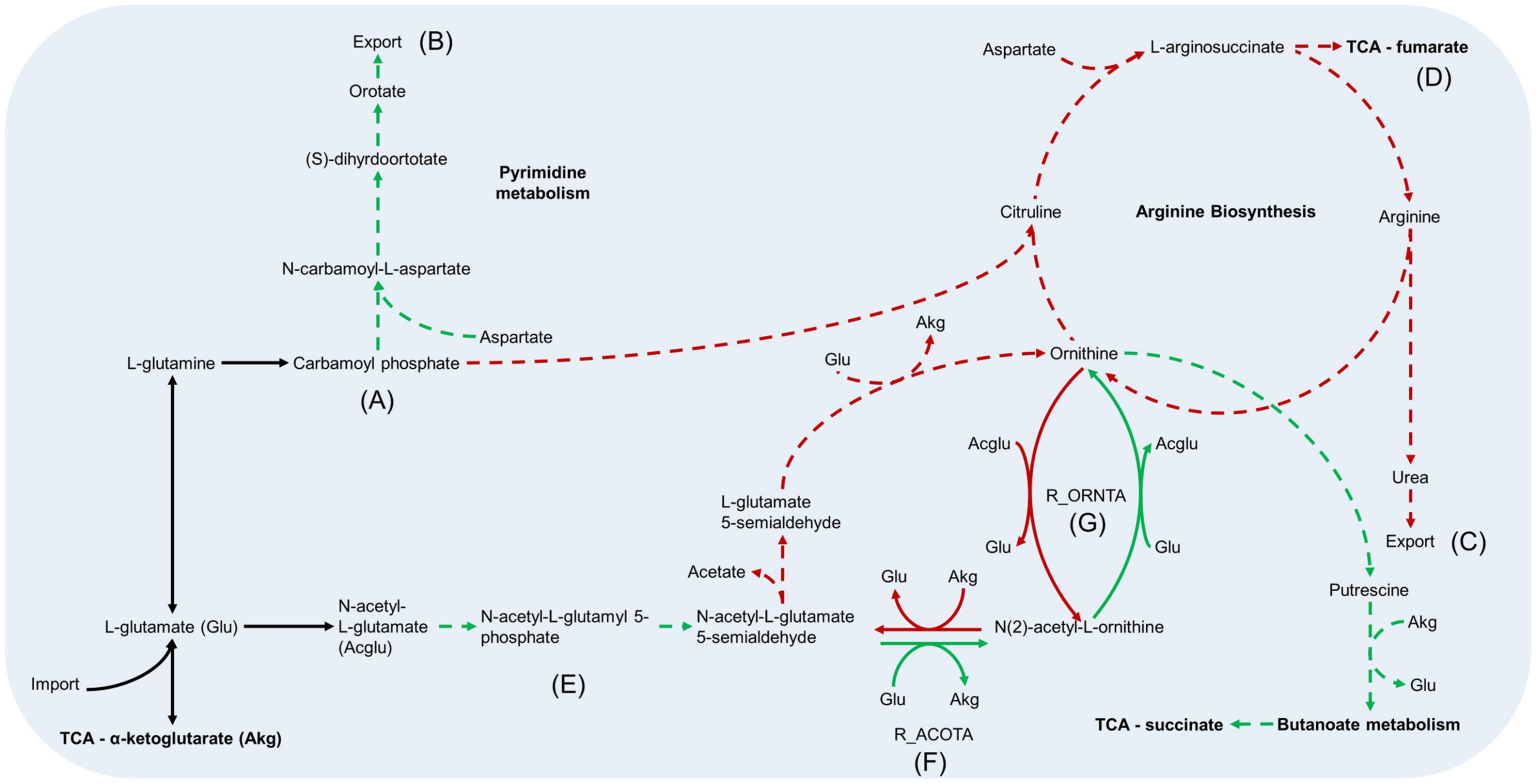
Arginine metabolism pathways in *B. pertussis* biofilm and planktonic iMAT models. This figure relates to Figure 5B. Models were generated using proteomic expression data. Green arrows represent reactions that are upregulated in biofilm cells while red arrows are downregulated reactions. Green dashed arrows are unique reactions to the biofilm model while red dashed arrows are reactions unique to the planktonic model. Black arrows are common reactions. General metabolic pathways are in bold. ⍺-ketoglutarate (Akg) and glutamate (Glu) are utilised in many of the reactions and are therefore included multiple times in abbreviated form. N-acetyl-L-glutamate is also included as the abbreviation Acglu for the reaction R_ORNTA. Key sections of the pathways have also been annotated. **(A)** Both models generate carbamoyl phosphate through the same reactions. **(B)** The biofilm model has unique reactions to synthesize orotate from carbamoyl phosphate and export it out the cell. **(C)** The planktonic model completed the arginine biosynthesis pathway and exports urea out of the cell. **(D)** In addition to the urea, the arginine biosynthesis pathway leads to the production of fumarate which is fed back into the TCA. **(E)** The pathways from N-acetyl-L-glutamate to N-acetyl-L-glutamate 5 semialdehyde are unique to the biofilm model. **(F)** The reaction, acetylornithine transaminase reaction (R_ACOTA), runs in opposite directions for planktonic and biofilm models. **(G)** The reaction, glutamate N-acetyl transferase (R_ORNTA), also runs in opposite directions for biofilm and planktonic models.

**Figure 8:**
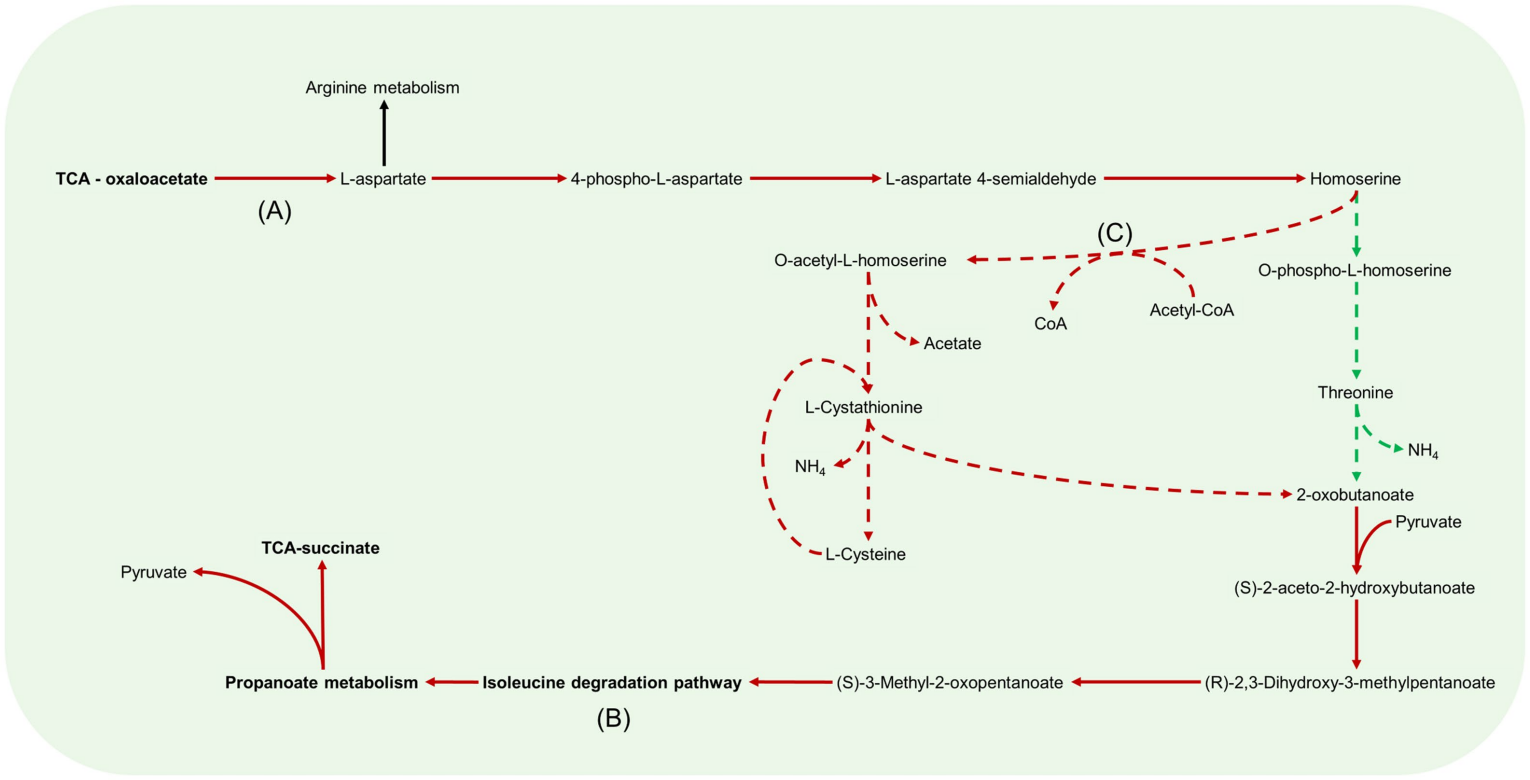
Aspartate metabolism pathways in *B. pertussis* biofilm and planktonic iMAT models. This figure relates to Figure 5C. Models were generated using proteomic expression data. Red arrows represent downregulated reactions in the biofilm model. Green dashed arrows are unique reactions to the biofilm model while red dashed arrows are reactions unique to the planktonic model. Black arrows are common reactions. General metabolic pathways are in bold. Key sections of the pathways have also been annotated. **(A)** There is a decreased flux from oxaloacetate to L-aspartate in the biofilm model. **(B)** In both models, 2-oxobutanoate is eventually degraded through the branched chain amino acid degradation pathway specifically, the isoleucine pathway. **(C)** While biofilm cells convert homoserine through to O-phospho-L-homoserine, planktonic cells convert homoserine to O-acetyl-L-homoserine by utilizing acetyl-CoA.

For an in-depth analysis of the metabolic changes between biofilm and planktonic cells, iMAT metabolic models were generated based on protein expression from both conditions. Of the 571 proteins identified using mass spectrometry, 228 were annotated with GPR associations in the *B. pertussis* (iBP1870) model (Branco Dos Santos et al., 2017). These are proteins which have assigned reactions in the base model. The overlap between the identified proteins and the reactions in the model constitutes 29.61% of the total GPR associations listed in the base model. Context specific models were created using the iMAT algorithm implemented in the COBRA Toolbox (Heirendt et al., 2019). The biofilm iMAT model consisted of 198 metabolites, 206 reactions and 188 genes. The planktonic iMAT model had 213 metabolites, 219 reactions and 195 genes (Table 2). To identify changes between the planktonic and biofilm iMAT models, the number of unique and common reactions between the two models were compared. There were 168 reactions that were common between the two models, 51 reactions that were unique to the planktonic model and 38 that were unique to the biofilm model (Figure 4A). The reactions were grouped into subsystems and total number of reactions in each group were similar between the two models (Figure 4B). It is notable however that there was a high proportion of reactions that were unique to the individual models. Major pathways are summarised and represented in Figure 5. Additionally, metabolic pathways for the TCA cycle, arginine metabolism, aspartate metabolism and glycerophospholipid metabolism pathways are highlighted in Figures 6–8.

**Table 2:** Number of metabolites, reactions and genes in the iMAT context specific models for *B. pertussis* biofilm and planktonic cells.

	Biofilm	Planktonic
High expression (+1)	36	36
Moderate expression (0)	117	86
Low expression (−1)	65	78
Total proteins incorporated	218	200
iMAT models		
Metabolites	198	213
Reactions	206	219
Genes	188	195

### *Bordetella pertussis* biofilm model completes the tricarboxylic acid cycle

An FBA was performed to predict potential changes in flux between reactions that were common between the planktonic and biofilm models. A major difference was seen in the TCA cycle. It was predicted that biofilm cells pushed flux to complete the TCA cycle while the planktonic model pushed flux toward the glyoxylate shunt (Figure 5A). While there was slightly higher levels of flux from oxaloacetate through to isocitrate in the planktonic model (planktonic: 10.40 mmol · g_DCW_^−1^ · h^−1^, biofilm: 8.92 mmol · g_DCW_^−1^ · h^−1^), the planktonic model pushed all flux (11.31 mmol · g_DCW_^−1^ · h^−1^) into the glyoxylate shunt to convert isocitrate to glyoxylate and succinate while comparatively, only a small flux (0.57 mmol · g_DCW_^−1^ · h^−1^) was predicted for the same reactions for the biofilm model (Figure 6). Instead, the flux moved toward ⍺-ketoglutarate (AKG) to succinyl-CoA and succinate to complete the TCA cycle (Figure 6). The planktonic model comparatively predicted no flux through these reactions. Despite these changes, there was an increased level of flux from succinate to fumarate and malate in the planktonic model which was fed from the glyoxylate shunt, branched chain amino acid degradation and the arginine biosynthesis cycle. Finally, there was also increased conversion of pyruvate to acetyl-CoA in the planktonic model which was then fed back into the TCA cycle as citrate. Most of the acetyl-CoA synthesis/utilisation pathways were either unique to the planktonic model or downregulated in the biofilm model. However, the biofilm model had unique reactions to convert acetyl-CoA to polyhydroxybutyrate (PHB) (Figure 5).

### Cardiolipin synthesis reactions present in the planktonic model

There were many reactions included in both models that were grouped into the glycerophospholipid metabolism subsystem. Flux was predicted through the gluconeogenesis pathway in both the models from pyruvate through to dihydroxyacetone phosphate and then glycerol 3-phosphate (Figure 5D). The pathways for most of the glycerophospholipids and fatty acid synthesis were the same between the two models. However, the planktonic model had pathways producing cardiolipin (tetradodecanoyl, n-C12:0) while the biofilm model lacked these pathways. The production of cardiolipin is through the pathway of conversion of 1,2-didodecanoyl-sn-glycero-3-cytidine 5′-diphosphate (CDP-DAG) to phosphatidylglycerophosphate (didodecanoyl, n-C12:0) and therefore there is a higher level of flux from the cytidine monophosphate (CMP) to cytidine triphosphate (CTP) in the planktonic model. The models suggest that there would be a higher level of 1-dodecanoyl-*sn*-glycerol 3-phosphate, 1-hexadec-9-enoyl-*sn*-glycerol 3-phosphate and glycerol 3-phosphate in the periplasm of the biofilm cells. In the planktonic model, it was predicted that there would be higher activity of reactions related to cardiolipin, phosphatidylglycerol (didodecanoyl, n-C12:0), 2-dodecanoyl-*sn*-glycerol 3-phosphate and 2-hexadec-9-enoyl-*sn*-glycerol 3-phosphate. The biofilm model had unique reactions to export glycerol 3-phosphate into the extracellular space through these pathways while planktonic model had predicted reactions that exported glycerol into the extracellular space (Figure 5D).

### Decreased arginine biosynthesis activity in the biofilm model

The metabolic models also revealed potentially altered amino acid metabolism pathways. There was an equal number of reactions grouped into the arginine and proline metabolism subsystem between the two models. However, the processes within the pathways were varied as seen by the number of unique reactions within the subsystem (Figure 4B). Both models predicted movement into the arginine metabolism pathways through conversion of L-glutamate (GLU). Subsequently, both models have reactions to produce carbamoyl phosphate (CBP) (Figure 7A). While the planktonic model pushed the CBP into the arginine biosynthesis pathway, the biofilm model had reactions to convert CBP through to orotate which was then exported out of the cell (Figure 7B). The reactions surrounding orotate were grouped in the purine and pyrimidine metabolism subsystem, which were predicted to be unique to the biofilm model (Figure 4B). The planktonic model completed the arginine biosynthesis cycle, with reactions exporting urea out of the cell as a by-product (Figure 7C). Furthermore, in the planktonic model, argininosuccinate is converted to arginine and fumarate which is fed back into the TCA cycle (Figures 5A, 7D). When each of these reactions were deleted in turn and tested for model feasibility, it led to infeasible models for the planktonic model but were still feasible for the biofilm model (Supplementary Table S3).

Both models were predicted to produce ornithine surrounding the arginine metabolism pathways. The planktonic model produced ornithine through the arginine biosynthesis pathway while the biofilm model synthesised ornithine through N-acetyl-L-glutamate to acetyl-ornithine and then to ornithine (Figure 7E). The fate of ornithine also differed greatly between the two models. When the FVA values were compared, the smallest Jaccard index value (lowest similarity) between the models were for the reactions acetylornithine transaminase (R_ACOTA) and glutamate N-acetyl transferase (R_ORNTA). R_ACOTA is a reversible reaction of acetyl-ornithine and AKG to N-acetyl-L-glutamate 5-semialdehyde and GLU. The FVA revealed that the biofilm model only had capabilities to run this reaction in reverse (Figure 7F). The opposite was seen for R_ORNTA, a reversible reaction of acetyl-ornithine and GLU to ornithine and N-acetyl-L-glutamate (Figure 7G). The planktonic model ran this reaction in reverse. Overall, the ornithine in the planktonic model was either utilised to produce acetate or pushed back through the arginine biosynthesis pathway. The biofilm model pushed ornithine through the butanoate metabolism pathway into the TCA cycle as succinate with the production of NADPH and NADH. This process utilised AKG and produced GLU.

### Downregulated aspartate metabolism in the biofilm model

Linked with the arginine metabolism pathways was the amino acid, aspartate (Figure 5C). Aspartate is synthesised in the model through the aspartate transaminase reaction which converts oxaloacetate and GLU to L-aspartate and AKG. This reaction had decreased predicted flux in the biofilm model (Figure 8A). Interestingly, when the reaction was deleted in the planktonic model, it led to an infeasible model while the biofilm model remained feasible without the reaction. The predicted flux in the biofilm model pushed from aspartate through to threonine synthesis and CBP metabolism. Threonine was eventually degraded through the branched chain amino acid (BCAA) degradation pathway (Figure 8B). Additionally, aspartate was uniquely converted with CBP to N-carbamoyl-L-aspartate and ultimately into orotate as mentioned above for the biofilm model (Figure 8B). The planktonic model had flux flow from aspartate to homoserine, but rather than following through to threonine, the homoserine was converted to O-acetyl-L-homoserine toward acetate and L-cystathionine (Figure 8C). L-cystathionine was then converted to L-cysteine and 2-oxobutanoate which was fed through the BCAA (specifically the isoleucine pathway) degradation pathway that was upregulated in the planktonic model. The BCAA degradation pathway leads to the production of acetyl-CoA, NADH, NADPH and FADH_2_. There was further production of these molecules through the valine degradation pathway in the planktonic model. These pathways led flux back into the TCA cycle as succinate and pyruvate through propanoate metabolism. Although the planktonic model moved flux through the valine degradation pathway, the reactions for the production of valine were unique to the biofilm model. The valine was either exported out of the cell or converted to AKG in the biofilm model.

Cysteine was predicted to be transported into the cell at the same rate between the two models. Both models source cysteine through diffusion, active ABC transport and the conversion of glutathione and L-cysteinylglycine. The biofilm model pushed all flux of cysteine to L-alanine while the planktonic model also has pathways to convert the cysteine through to acetyl-CoA as mentioned above (Figure 8C). The reactions to move L-alanine in and out of the periplasm were unique to the biofilm model. The main source of L-alanine for the biofilm cells was from the conversion of pyruvate and glutamine to L-alanine and AKG. The planktonic model creates L-alanine through the conversion of β-alanine. This reaction also creates malonate-semialdehyde and was strongly downregulated in the biofilm model. The L-alanine was converted to D-alanine and then to GLU and pyruvate in both models.

### Increased superoxide dismutase activity in the biofilm model

*Bordetella pertussis* has traditionally been classified as an aerobic organism with oxygen as its preferred terminal electron acceptor (Wan et al., 2009). There are limited studies on the effect of oxygen variation on *B. pertussis* growth. In the present study, it was predicted that the biofilm model had a slightly lower uptake of oxygen compared to the planktonic model and this may be linked to decreased activity of the electron transport chain. Both models converted ubiquinol to ubiquinone through cytochrome ubiquinol oxidase. This reaction was downregulated in biofilm model. However, the biofilm model also had an additional reaction that converted ubiquinol to ubiquinone with the by-product of superoxide anions. The superoxide was converted to hydrogen peroxide and O_2_ by superoxide dismutase. The hydrogen peroxide was converted to H_2_O by thioredoxin. The other reactions involving ubiquinone were downregulated in biofilm cells including succinate dehydrogenase and NADH dehydrogenase. However, ubiquinone was converted to ubiquinol through the conversion S-dihydroorotate to orotate in a reaction unique to the biofilm cells.

### Comparison with other metabolic models

We further used proteomics data from a previous *B. pertussis* biofilm study by de Gouw et al. (2014) to create iMAT models and compare the reactions. The study by de Gouw et al. (2014) grew *B. pertussis* biofilms on flat polypropylene beads in a glass column reactor for 72 h with THIJS media refreshed every 24 h. The planktonic cells were extracted at both mid-exponential phase at 17 h and stationary phase at 40 h. That study identified 729–825 proteins from the three different conditions with an overlap of 645 proteins (de Gouw et al., 2014). While the biofilm iMAT model generated using that proteomic data had a higher number of reactions, metabolites and genes to the planktonic iMAT model generated from that same data, the overall values were comparable to the models of this study (Supplementary Table S1).

In line with our study, the de Gouw et al. (2014) biofilm model predicted completion of the full TCA cycle while the planktonic model pushed flux through the glyoxylate shunt. Further similarities were identified between the biofilm models in NADH dehydrogenase, aspartate transaminase (Figure 8A) and CO_2_ and O_2_ exchange. All biofilm models had decreased flux in these reactions compared to their planktonic counterpart. The NADH dehydrogenase reaction produced NAD+ from NADH with the conversion of ubiquinone to ubiquinol. The aspartate transaminase reaction (R_ASPTA – reversed) had decreased flux for all biofilm models. This reaction converted oxaloacetate and GLU to AKG and L-aspartate. The reaction from aspartate to CBP and subsequent reactions to orotate were unique to the biofilm models (Figure 7B; Supplementary Table S3).

## Discussion

Recent studies have shown that biofilms are an important aspect of *B. pertussis* pathogenesis (Bosch et al., 2005; Arnal et al., 2015; Cattelan et al., 2018). Biofilm cells are more resilient against antimicrobials and environmental stresses (Mishra et al., 2005; Dorji et al., 2016). While previous studies of *B. pertussis* biofilms have made important discoveries related to growth and virulence, there has been less research focused on biofilm metabolism. Biofilms are readily formed by *B. pertussis* and has been defined as an integral part of its pathogenesis (Conover et al., 2010). Investigating the metabolic changes that occur within a biofilm community may help increase the understanding of *B. pertussis* adaption to the host (Holban et al., 2022). Therefore, this study integrated proteomic expression data from a currently circulating epidemic strain into a metabolic model of *B. pertussis* to identify major changes that occur in metabolism between the biofilm and planktonic states. To our knowledge this is the first extensive study into specific metabolic pathways within biofilms of *B. pertussis*. Although utilised metabolic pathways differ from species to species, many of the changes in this study have been identified and confirmed experimentally in other species reinforcing the strength of the metabolic models. The major metabolic differences predicted in this study relate to the TCA cycle, amino acid metabolism and virulence.

The TCA cycle is the major central metabolic pathway for many aerobic organisms, therefore it was surprising to find that there was altered flux for the TCA cycle. There was a major predicted shift for the planktonic model to utilise the glyoxylate shunt instead of completing the full TCA cycle. A recent study (Anziani et al., 2023), performed a temporal multi-omics analysis on planktonic *B. pertussis* cells and identified that the genes and proteins involved in the TCA cycle from the conversion of ⍺-ketoglutarate to fumarate had relatively low activity until after 12 h. These reactions would have lower activity during activation of the glyoxylate shunt reinforcing the flux predicted in this study. Additionally, it was reported that the proteins and genes involved in glyoxylate metabolism decreased following 12 h before increasing again after 18 h and 45 min (Anziani et al., 2023). The extracellular metabolites that were measured also reinforced this trend (Anziani et al., 2023). Variation in the glyoxylate shunt has been observed in the biofilms of other species. When the glyoxylate shunt was disabled in *Pseudomonas aeruginosa*, there was an increase in biofilm formation (Ahn et al., 2016), which is reflected in this study. It was suggested that the increased extracellular polymeric substances (EPS) produced when the glyoxylate shunt was disabled could lead to higher survival in the microaerobic conditions of the cystic fibrosis lung environment (Ahn et al., 2016). Additionally, there was an increased level of glyoxylate activity in *Candida albicans* cells dispersed from biofilm (Uppuluri et al., 2018). It is hypothesised that as the glyoxylate shunt is activated to increase nutrient versatility, it may be an anticipatory reaction for low nutrient levels while searching for a new colonisation location (Uppuluri et al., 2018). The dispersed cells may reflect a planktonic lifestyle while established mature biofilm cells may utilise the network of cells to share nutrients and hence have a decreased requirement of the glyoxylate shunt. Targeting the TCA cycle has been suggested as a potential therapeutic strategy against biofilms (Yahya et al., 2014; Arnal et al., 2015). Additional models that were created using *B. pertussis* biofilm and planktonic proteomic expression data from de Gouw et al. (2014) had similar differences in the TCA cycle to our results (Supplementary Figure S2).

Increased polyhydroxybutyrate (PHB) and superoxide dismutase activity may explain the increased survivability of *B. pertussis* biofilm cells. Reactions regarding PHB synthesis were predicted as unique to the biofilm model and superoxide dismutase activity was potentially increased in the biofilm model. While most reactions surrounding acetyl-CoA were downregulated in the biofilm model or unique in the planktonic model, a set of reactions that involve the conversion of acetyl-CoA to PHB were uniquely predicted in both the biofilm models. The PHB reactions would lead to an increase in cytoplasmic PHB demand. It has been previously reported that cytoplasmic PHB inclusions exist in *B. pertussis* cells (Thalen et al., 1999). These inclusions are generated when there are high levels of carbon in the environment or when *B. pertussis* cells are undergoing iron starvation (Thalen et al., 1999; Alvarez Hayes et al., 2015). It has been hypothesised that the cells generate PHB inclusions as an energy reserve for when cells are in harsh environments (Thalen et al., 1999). As the PHB demand reactions are increased in the biofilm cells, this may lead to increased cell survivability that has been observed in *B. pertussis* biofilms (Dorji et al., 2016). Furthermore, the predicted increase in the superoxide dismutase activity may be protective as it leads to a decrease in superoxide which, as a free radical, may lead to cellular damage (De Groote et al., 1997; Suo et al., 2012). Put together, the models highlighted that these two factors may be related to the increased cell survivability of *B. pertussis* biofilm cells and may lead to persistent infections.

Strongly linked with nutrient acquisition and metabolism, there were major expression differences identified for virulence factors. Prn was found to be downregulated in biofilm cells. This is contrasted by previous results that have found an increase in Prn in *B. pertussis* biofilm cells (Serra et al., 2008; de Gouw et al., 2014; Arnal et al., 2015; Carriquiriborde et al., 2021). However, it should be noted that de Gouw et al. had slightly decreased levels of Prn in biofilm cells compared to mid-log planktonic cells while there was a small upregulation compared to stationary phase cells (de Gouw et al., 2014). As this current study compared with early log phase cells, the difference in Prn may be related to variations in planktonic or biofilm phases of growth. Nevertheless, to our knowledge, this is the first report of a major downregulation of Prn in *B. pertussis* biofilm cells. As one of the three ACV components, the change in Prn identified supports the utility of biofilm related proteins as novel vaccine antigens to better target biofilm cells *in vivo* (de Gouw et al., 2014; Dorji et al., 2019; Carriquiriborde et al., 2021). Additionally, another ACV component, Ptx had varied expression dependent of the subunit. PtxA was downregulated in biofilm cells while PtxD was found uniquely in biofilm cells. As it has previously been reported that the subunits can have independent immune modularly activity, the variation in Ptx subunit expression may be indicative of distinct immune evasion approaches between the two conditions (Mangmool and Kurose, 2011). This study provides additional targets that may be explored further in addition to identifying key metabolic pathways that may be crucial to disrupting the biofilm lifestyle.

The virulence factor, adenylate cyclase toxin, CyaA, was upregulated in the biofilm cells. CyaA has been shown to interact with FHA and decrease biofilm formation in a concentration dependent manner (Hoffman et al., 2017). Furthermore, it was found that the addition of exogenous CyaA can lead to the diffusion of preformed *B. pertussis* biofilms (Hoffman et al., 2017). It has been shown that beyond 96 h of incubation the rate of *B. pertussis* biofilm formation can plateau (Serra et al., 2008). It is possible that *B. pertussis* utilises the activity of CyaA to diffuse cells from the biofilm to control growth in addition to allowing the spread of biofilm cells.

Traditional analysis of proteomic data to infer metabolic changes involves extrapolating metabolic activity based on individual protein expression, however, the level of protein abundance is not always directly proportional to enzymatic activity. Therefore, in this study, protein expression data was used as cues to assess the likelihood of the activity of a particular metabolic pathway. The major advantage of this approach is that it narrows down the highly extensive network of reactions in the GSMM to predict pathways that are potentially context specific. The incorporation of the proteomic data in the GSMM in this study provided new insights into the metabolic pathways that are potentially altered between biofilm and planktonic cells. Further experimental studies confirming the changes in metabolism would increase confidence in the results identified. Nevertheless, an initial glimpse into the potentially active metabolic pathways within *B. pertussis* biofilm cells was established. Parallels with other organisms that have similar fluctuations in biofilm pathways reinforce the value of the models and help validate the predicted changes identified.

It is important to note that the biofilm cells were grown in an artificial media (THIJS media) designed to optimise *B. pertussis* growth (Thalen et al., 1999). Thus, the metabolic models may be reflective of growth specifically in this medium. Furthermore, as the media was not refreshed, there would be a decrease in the available nutrients over time. It would be interesting to see how growth in different media such as media more representative of the nutrients available in the respiratory environment or co-culture with epithelial cells would affect the metabolic models (Palmer et al., 2007). Additionally, the proteomic data measures the average protein expression independent of space and time. Biofilms are heterogeneous communities with gradients in nutrient diffusion and distinct developmental stages (Patel and Bott, 1991; Stewart, 2003; Stewart et al., 2016; Díaz-Pascual et al., 2021). Additional spatial and temporal metabolic models will further identify changes that occur throughout biofilm development (Siriwach et al., 2020). The de Gouw et al. (2014) proteomic data was extracted at 72 h compared to 96 h in this study and may represent biofilms at different stages of development. Finally, planktonic cells in the log phase were used for the comparison, there may be more similarities between the two conditions when planktonic cells are grown for a longer period in stationary phase.

In conclusion, this study compared the proteomic expression of biofilm and planktonic *B. pertussis* cells and identified key changes between the conditions including an upregulation of toxins (adenylate cyclase toxin and dermonecrotic toxin) and downregulation of pertactin and type III secretion system proteins in biofilm cells. Incorporation of proteomic data into a genome scale metabolic model predicted major metabolic changes that may occur during biofilm conditions in *B. pertussis*. Notably, it was predicted that the biofilm model utilised the full TCA cycle while the planktonic model pushed flux through the glyoxylate shunt. There was a predicted increase in PHB accumulation and superoxide dismutase activity which may lead to increased persistence of biofilm cells. Our study highlights the utility of integrating expression data into metabolic modeling. Overall, the changes identified in this study helps lay the groundwork for further studies into *B. pertussis* biofilms and its role in pathogenesis.

## Data availability statement

The datasets presented in this study can be found in online repositories. The names of the repository/repositories and accession number(s) can be found in the article/Supplementary material.

## Author contributions

HS, LL, and RL designed the study. LL, LZ, MR, and RL provided critical analysis and discussion. HS wrote the first draft. All authors contributed to the final manuscript.

## Funding

This work was funded by a project grant from the National Health and Medical Research Council of Australia (grant number 1146938). HS was supported by an Australian Government Research Training Program (RTP) Scholarship. The funders had no role in study design, data collection and interpretation, or the decision to submit the work for publication.

## Conflict of interest

The authors declare that the research was conducted in the absence of any commercial or financial relationships that could be construed as a potential conflict of interest.

## Publisher’s note

All claims expressed in this article are solely those of the authors and do not necessarily represent those of their affiliated organizations, or those of the publisher, the editors and the reviewers. Any product that may be evaluated in this article, or claim that may be made by its manufacturer, is not guaranteed or endorsed by the publisher.
