# Supplementary material for: Integrating proteomic data with metabolic modeling provides insight into key pathways of Bordetella pertussis biofilms
Source: Front Microbiol. 2023 Aug 3;14:1169870. doi: 10.3389/fmicb.2023.1169870 (PMC10435875; doi:10.3389/fmicb.2023.1169870)
Supplement: Supplementary file 2 [file Data_Sheet_2.docx]

**Supplementary materials**

**Supplementary figures and tables**

A


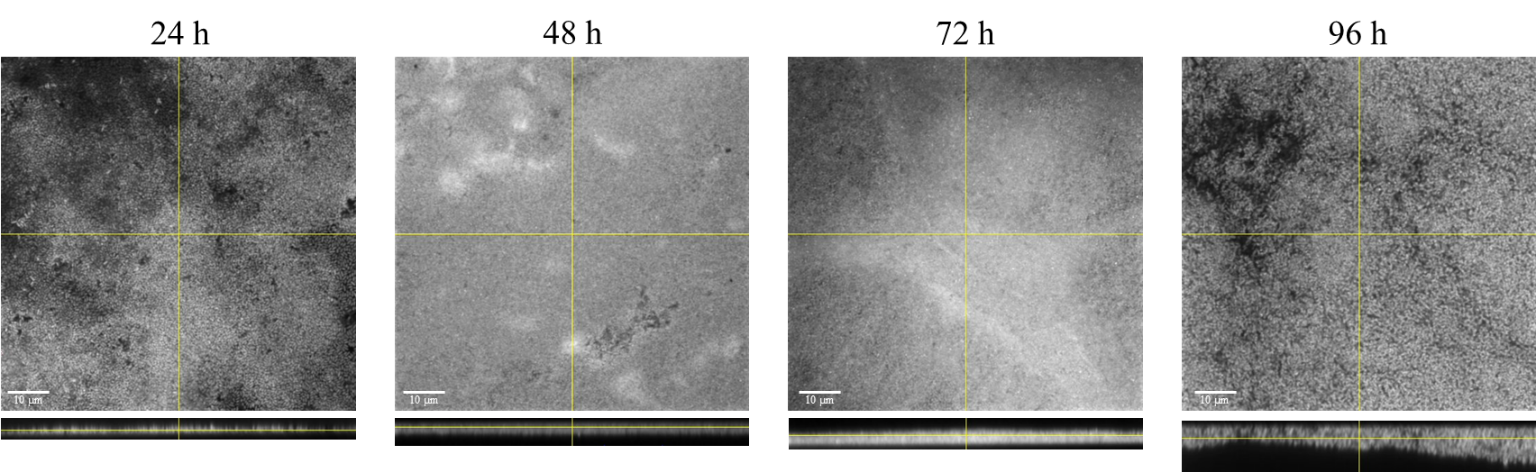


B

C

**Supplementary Figure S1** **A** Growth curve of L1423 planktonic cells. OD_600nm_ was measured every 12 h until 60 h. At each timepoint 100 µL of culture was removed and a serial dilution was performed in PBS before being plated on BG agar. BG agar plates were incubated for 5 days at 37°C. Three biological replicates were performed. **B** Confocal laser scanning microscopy micrographs of currently circulating strain, L1423 *B. pertussis* biofilm formation from 24 h to 96 h. Biofilms were grown on glass cover slides imaged in a z-stack setting. Images are presented in xy and xz planes. Images are represented in colourblind-safe grayscale (1). **C** Biomass calculated at each time point using the COMSTAT2 ImageJ plugin. Three biological replicates per time point were performed with 3 randomly selected field of views for extracted values.

**Supplementary Figure S2 Flux values for reactions in the tricarboxylic acid cycle between additional models.** Reactions extracted from FBA results for the models in this study (biofilm and planktonic) and de Gouw *et al.* (biofilm and planktonic) (2). R_CS, type II citrate synthase; R_ACONTa, citrate hydrolase; R_ACONTb, aconitate hydratase; R_ICDHyr, isocitrate dehydrogenase; R_AKGDH, α-ketoglutarate dehydrogenase; R_SUCOAS, succinyl-CoA synthetase; R_SUCDi, succinate dehydrogenase; R_FUM, fumarate hydratase; R_MDH, malate dehydrogenase; R_ICL, isocitrate lyase; R_MALS, malate synthase.

**Supplementary Table S1** Comparison of conditions used in the other studies from which additional iMAT models were extracted. Included are the number of reactions, metabolites and genes in each metabolic model.

|  | This study biofilm | This study planktonic | de Gouw biofilm (2) | de Gouw planktonic (2) |
| --- | --- | --- | --- | --- |
| Media | THIJS | THIJS | THIJS | THIJS |
| Incubation time | 96 h | 12 h | 72 h | 17 h/40 h |
| Strain | L1423 | L1423 | B1917 | B1917 |
| Vessel | 24 well plate | 20 mL Bioreactor tube | Polypropylene beads | 500 mL Erlenmeyer flasks |
| Media refreshment | 5 hr | NA | 5 hr then every 24 hr | NA |
| **iMAT models** |  |  |  |  |
| Reactions | 206 | 219 | 194 | 134 |
| Metabolites | 198 | 213 | 191 | 127 |
| Genes | 188 | 195 | 195 | 150 |

**Supplementary discussion**

**Growth curve of *B. pertussis* biofilms**

The growth of the *B. pertussis* biofilm represented in Supplementary Figure S1 showed steady biomass formation up to 72 h before an increase in biomass to 96 h. This may be indicative of an induction phase before transitioning to an exponential biofilm phase that has been previously reported for strong biofilm formers (3). This is further indication that L1423 has strong biofilm forming capabilities that are likely to have relevance to infection (4).

**Strain differences between de Gouw *et al.* (2) study and this study**

The strains used by de Gouw *et al*. (2) and this study are both of the same genotype *ptxP3-ptxA1-prn2*. The *ptxP3* strains have overtaken the previously dominant *ptxP1* strains and have been linked to the resurgence in pertussis cases (5). However, there are some differences between the strains. B1917 was isolated from a Dutch patient in 2000 while L1423 was isolated in Australia during the 2008 – 2012 epidemic (6). The genomes of the strains have been previously compared and found to have 11 non-synonymous SNPs and 6 indels between them (7). These variations may account for the differences identified between the two studies. Furthermore, there are differences in the growth conditions that are described in **Supplementary Table S1**.

**Cardiolipin metabolism**

Many pathways for glycerophospholipid metabolism were predicted to be active in both metabolic models. Lipid remodelling can occur when cells are exposed to harsh environments or antimicrobials (8). The changes in the lipids have been linked to increased survivability and persistence in pathogenic bacterial biofilms (9). The models in this study hint at potentially differing levels of certain glycerophospholipids in the periplasm between planktonic and biofilm cells. Of particular interest is cardiolipin, predicted as a unique lipid synthesis pathway in the planktonic model. Previous studies have shown that cardiolipin depletion led to decreased biofilm in *E. coli* (10). Cardiolipins have been identified in *B. pertussis* previously however, studies have not remarked on biofilm cells (11). It is important to note that the proteomic data captures enzymes that are present at a particular time point rather than a measurement of lipids, therefore, the changes identified in these reactions merely provide a snapshot of the time from which the proteins were harvested. The planktonic cells may continuously synthesise cardiolipin while mature biofilm cells either synthesise cardiolipin at an earlier stage of biofilm or have a lower requirement for cardiolipin. To further investigate the changes in lipid composition, future lipidomic analysis of biofilm cells would be necessary. Nevertheless, the metabolic models in this study predicted differing lipid compositions in biofilm and planktonic *B. pertussis* cell membranes. Another interesting change that was linked to the cardiolipin pathway was the exportation of glycerol out of the cell. While the planktonic model was predicted to export glycerol, the biofilm model has reactions to export glycerol 3-phosphate. The reason for this is unexplored and it would be interesting to identify whether varying concentrations of glycerol 3-phosphate or glycerol would have a regulatory role in *B. pertussis* (12).

**Amino acid metabolism**

Many regulatory processes are involved in *B. pertussis* biofilm formation (4). This study predicted multiple amino acid processing pathways that varied between biofilm and planktonic models. It has been reported previously that amino acids and their intermediates can act as regulatory molecules (13-15). The planktonic cells showed increased arginine biosynthesis and aspartate transaminase activity. It has been shown in other species that arginine may increase biofilm formation although higher concentrations have an inhibitory effect (14) and increase antibiotic killing (16). In addition, arginine may disrupt bacterial coaggregation to prevent biofilm formation (17). Finally, the arginine biosynthesis process leads to urea by-products which have been previously shown to disrupt the EPS matrix of biofilms (18). The evidence in these studies combined with results from this present study points to a tightly regulated arginine synthesis pathway which is partially fed by the aspartate transaminase reaction. While the planktonic model was predicted to push aspartate to the arginine biosynthesis pathway, the biofilm model had reactions to push aspartate to the threonine degradation pathway and towards the synthesis of N-carbamoyl-L-aspartate (through to orotate) leading to an overall reduction of available arginine. There were also reactions to export orotate out of the biofilm cells rather than process orotate for pyrimidine metabolism. Orotate therefore may have the potential to act as a signalling molecule for *B. pertussis* biofilm cells. The pathways regarding carbamoyl phosphate synthesis which leads to orotate synthesis has been shown to be altered in biofilm formation in other species (19, 20). This orotate production pathway was predicted to be specific to both the biofilm model in this study and the biofilm model created from the data of de Gouw *et al.* (2), reinforcing the possible importance of orotate for *B. pertussis* biofilms.

Within the process to synthesise orotate is the incorporation of aspartate to carbamoyl phosphate (Figure 7A). Aspartate conversion from oxaloacetate and GLU (aspartate transaminase reaction) was predicted to be a downregulated pathway in both biofilm metabolic models compared to their planktonic counterparts. Aspartate has been seen to inhibit biofilm formation in multiple *Staphylococcus* species (21). Furthermore, the same specific aspartate transaminase reaction was downregulated in *Streptococcus pneumoniae* biofilms (22). Thus, it is likely that aspartate may also have an inhibitory effect on *B. pertussis* biofilms.

Finally, there was a predicted change in the processing of alanine and valine between the biofilm and planktonic models. The models suggest that β-alanine is important for planktonic cells while L-alanine is more vital for biofilm cells. The biofilm model had unique reactions transporting L-alanine into the periplasm. A recent study showed that alanine metabolism activity displays a unique pattern based on spatial distribution of cells within a heterogenous biofilm (23). The alanine metabolism changes identified in the present study may relate to a particular layer of the biofilm. It would be interesting to see the specific active reactions between the layers of biofilm. Proteomic analysis on layers of biofilm have previously been performed using laser ablation sample transfer (LAST) and label free quantification mass spectrometry (24). A combination of LAST and the methods used in this study may be able to confirm the changes in alanine metabolism. Furthermore, valine was predicted to be uniquely exported out of the biofilm model. This reinforces what has been observed in other Gram-negative species that had increased valine secretion in biofilm conditions (25). It was suggested that within the low oxygen and reduced growth rate conditions of biofilms, there would be an excess of pyruvate leading to an increase in valine and alanine biosynthesis (25). The discussed changes in amino acid metabolism pathways are biofilm specific. It is possible that the secreted molecules could work as signalling molecules potentially triggering biofilm formation (13-15, 26).

**Bvg System**

The Bvg system has been shown to regulate most of the virulence factors in *B. pertussis* (27). This study identified increased expression of the BvgA regulator and BvgS in biofilms while the expression of BvgR was similar between the two conditions (Figure 2B and Table 1). Upregulation of BipA underscores the importance of the Bvg^i^ phase in the biofilm existence (2, 28). However, it should be noted that there were also changes in both Bvg^+^ and Bvg^-^ protein regulation. Unexpectedly, there was a wide variability in protein expression within Bvg regulated proteins. For example, in addition to varied levels between CyaA and Prn mentioned above, Dnt was upregulated while many proteins of the type III secretion system (T3SS) were downregulated. Within both the upregulated and downregulated proteins, 13-15% of the proteins were Bvg^+^. Furthermore, there was a significantly higher proportion (14%) of proteins upregulated in biofilms that were Bvg^-^ compared to the proportion (4%) of proteins downregulated that were Bvg^-^ (Fishers exact test, *p* = 0.0002). These findings suggest that there may be a heterogeneous mixture of Bvg mode cells distributed throughout the biofilm. As there are variations in the nutrients diffused throughout a biofilm (29, 30), it is rational to expect that phenotypic modulation would occur based on the nutrients available within the microenvironment for each given cell.

It has been shown that there are varying levels of diffusion of CO_2_ and O_2_ within a biofilm (30-32). *B. pertussis* encodes regulatory systems (PlrSR and *Bpe*GReg) that respond to fluctuations in concentrations of CO_2_ and O_2_ (33, 34). While *Bpe*GReg was not identified in this study, the PlrSR system was found to be upregulated in the biofilm cells and may be indicative of varying levels of CO_2_ within the biofilm. Furthermore, the PlrSR system may lead to the change in the expression of Bvg proteins measured in this study (33). There was a range of additional response and transcriptional regulators with differential expression in biofilm cells (Supplementary Table S2). The findings in this study point to other major regulatory systems that affect the regulation of virulence proteins in biofilm conditions.

**Supplementary references**

1. Johnson, J. 2012. Not seeing is not believing: improving the visibility of your fluorescence images. *Mol. Biol. Cell* 23: 754-757.

2. de Gouw, D., D. O. Serra, M. I. de Jonge, P. W. Hermans, H. J. Wessels, A. Zomer, O. M. Yantorno, D. A. Diavatopoulos, and F. R. Mooi. 2014. The vaccine potential of *Bordetella pertussis* biofilm-derived membrane proteins. *Emerg. Microbes Infect.* 3: e58.

3. Bruchmann, J., K. Sachsenheimer, B. E. Rapp, and T. Schwartz. 2015. Multi-Channel Microfluidic Biosensor Platform Applied for Online Monitoring and Screening of Biofilm Formation and Activity. *PLoS One* 10: e0117300.

4. Cattelan, N., P. Dubey, L. Arnal, O. M. Yantorno, and R. Deora. 2016. *Bordetella* biofilms: a lifestyle leading to persistent infections. *Pathog. Dis.* 74: ftv108.

5. Mooi, F. R., I. H. van Loo, M. van Gent, Q. He, M. J. Bart, K. J. Heuvelman, S. C. de Greeff, D. Diavatopoulos, P. Teunis, N. Nagelkerke, and J. Mertsola. 2009. *Bordetella pertussis* strains with increased toxin production associated with pertussis resurgence. *Emerg. Infect. Dis.* 15: 1206-1213.

6. Safarchi, A., S. Octavia, S. Z. Wu, S. Kaur, V. Sintchenko, G. L. Gilbert, N. Wood, P. McIntyre, H. Marshall, A. D. Keil, and R. Lan. 2016. Genomic dissection of Australian *Bordetella pertussis* isolates from the 2008–2012 epidemic. *J. Infect.* 72: 468-477.

7. Luu, L. D. W. 2018. Comparative proteomic analysis of Australian epidemic *Bordetella pertussis*. In *School of Biotechnology and Biomolecular Sciences*. University of New South Wales.

8. Seydlova, G., J. Beranova, I. Bibova, A. Dienstbier, J. Drzmisek, J. Masin, R. Fiser, I. Konopasek, and B. Vecerek. 2017. The extent of the temperature-induced membrane remodeling in two closely related *Bordetella* species reflects their adaptation to diverse environmental niches. *J. Biol. Chem.* 292: 8048-8058.

9. Dubois-Brissonnet, F., E. Trotier, and R. Briandet. 2016. The Biofilm Lifestyle Involves an Increase in Bacterial Membrane Saturated Fatty Acids. *Front. Microbiol.* 7: 1673.

10. Nepper, J. F., Y. C. Lin, and D. B. Weibel. 2019. Rcs Phosphorelay Activation in Cardiolipin-Deficient *Escherichia coli* Reduces Biofilm Formation. *J. Bacteriol.* 201: e00804-00818.

11. Kawai, Y., and A. Moribayashi. 1982. Characteristic lipids of *Bordetella pertussis*: simple fatty acid composition, hydroxy fatty acids, and an ornithine-containing lipid. *J. Bacteriol.* 151: 996-1005.

12. Wapnir, R. A., and L. Stiel. 1985. Regulation of gluconeogenesis by glycerol and its phosphorylated derivatives. *Biochem. Med.* 33: 141-148.

13. Cava, F., H. Lam, M. A. de Pedro, and M. K. Waldor. 2011. Emerging knowledge of regulatory roles of D-amino acids in bacteria. *Cell Mol. Life Sci.* 68: 817-831.

14. Goh, S.-N., A. Fernandez, S.-Z. Ang, W.-Y. Lau, D.-L. Ng, and E. S. G. Cheah. 2013. Effects of Different Amino Acids on Biofilm Growth, Swimming Motility and Twitching Motility in *Escherichia coli* BL21. *Journal of Biology and Life Science* 4: 13.

15. Aliashkevich, A., L. Alvarez, and F. Cava. 2018. New Insights Into the Mechanisms and Biological Roles of D-Amino Acids in Complex Eco-Systems. *Front. Microbiol.* 9: 683.

16. Borriello, G., L. Richards, G. D. Ehrlich, and P. S. Stewart. 2006. Arginine or Nitrate Enhances Antibiotic Susceptibility of *Pseudomonas aeruginosa* in Biofilms. *Antimicrob. Agents Chemother.* 50: 382-384.

17. Kolderman, E., D. Bettampadi, D. Samarian, S. E. Dowd, B. Foxman, N. S. Jakubovics, and A. H. Rickard. 2015. L-Arginine Destabilizes Oral Multi-Species Biofilm Communities Developed in Human Saliva. *PLoS One* 10: e0121835.

18. Brindle, E. R., D. A. Miller, and P. S. Stewart. 2011. Hydrodynamic deformation and removal of *Staphylococcus epidermidis* biofilms treated with urea, chlorhexidine, iron chloride, or DispersinB. *Biotechnol. Bioeng.* 108: 2968-2977.

19. Zhuo, T., W. Rou, X. Song, J. Guo, X. Fan, G. G. Kamau, and H. Zou. 2015. Molecular study on the *carAB* operon reveals that *carB* gene is required for swimming and biofilm formation in *Xanthomonas citri* subsp. *citri*. *BMC Microbiol.* 15: 225-225.

20. Pisithkul, T., J. W. Schroeder, E. A. Trujillo, P. Yeesin, D. M. Stevenson, T. Chaiamarit, J. J. Coon, J. D. Wang, D. Amador-Noguez, J.-M. Ghigo, and E. P. Greenberg. 2019. Metabolic Remodeling during Biofilm Development of *Bacillus subtilis*. *mBio* 10: e00623-00619.

21. Yang, H., M. Wang, J. Yu, and H. Wei. 2015. Aspartate inhibits *Staphylococcus aureus* biofilm formation. *FEMS Microbiol. Lett.* 362: fnv025.

22. Allan, R. N., P. Skipp, J. Jefferies, S. C. Clarke, S. N. Faust, L. Hall-Stoodley, and J. Webb. 2014. Pronounced metabolic changes in adaptation to biofilm growth by *Streptococcus pneumoniae*. *PLoS One* 9: e107015.

23. Díaz-Pascual, F., M. Lempp, K. Nosho, H. Jeckel, J. K. Jo, K. Neuhaus, R. Hartmann, E. Jelli, M. F. Hansen, A. Price-Whelan, L. E. P. Dietrich, H. Link, and K. Drescher. 2021. Spatial alanine metabolism determines local growth dynamics of *Escherichia coli* colonies. *eLife* 10: e70794.

24. Pulukkody, A. C., Y. P. Yung, F. Donnarumma, K. K. Murray, R. P. Carlson, and L. Hanley. 2021. Spatially resolved analysis of *Pseudomonas aeruginosa* biofilm proteomes measured by laser ablation sample transfer. *PLoS One* 16: e0250911.

25. Valle, J., S. Da Re, S. Schmid, D. Skurnik, R. D'Ari, and J. M. Ghigo. 2008. The amino acid valine is secreted in continuous-flow bacterial biofilms. *J. Bacteriol.* 190: 264-274.

26. Idrees, M., A. R. Mohammad, N. Karodia, and A. Rahman. 2020. Multimodal Role of Amino Acids in Microbial Control and Drug Development. *Antibiotics (Basel)* 9: 330.

27. Cotter, P. A., and A. M. Jones. 2003. Phosphorelay control of virulence gene expression in *Bordetella*. *Trends Microbiol.* 11: 367-373.

28. Deora, R. 2004. Multiple mechanisms of *bipA* gene regulation by the *Bordetella* BvgAS phosphorelay system. *Trends Microbiol.* 12: 63-65.

29. Salgar-Chaparro, S. J., K. Lepkova, T. Pojtanabuntoeng, A. Darwin, L. L. Machuca, and A. J. M. Stams. 2020. Nutrient Level Determines Biofilm Characteristics and Subsequent Impact on Microbial Corrosion and Biocide Effectiveness. *Appl. Environ. Microbiol.* 86: e02885-02819.

30. Stewart, P. S. 2003. Diffusion in biofilms. *J. Bacteriol.* 185: 1485-1491.

31. Stewart, P. S., T. Zhang, R. Xu, B. Pitts, M. C. Walters, F. Roe, J. Kikhney, and A. Moter. 2016. Reaction–diffusion theory explains hypoxia and heterogeneous growth within microbial biofilms associated with chronic infections. *npj Biofilms Microbomes* 2: 16012.

32. Patel, T. D., and T. R. Bott. 1991. Oxygen diffusion through a developing biofilm of *Pseudomonas fluorescens*. *J. Chem. Technol. Biotechnol.* 52: 187-199.

33. Bone, M. A., A. J. Wilk, A. I. Perault, S. A. Marlatt, E. V. Scheller, R. Anthouard, Q. Chen, S. Stibitz, P. A. Cotter, and S. M. Julio. 2017. *Bordetella* PlrSR regulatory system controls BvgAS activity and virulence in the lower respiratory tract. *Proc. Natl. Acad. Sci. U.S.A.* 114: E1519-E1527.

34. Wan, X., J. R. Tuckerman, J. A. Saito, T. A. K. Freitas, J. S. Newhouse, J. R. Denery, M. Y. Galperin, G. Gonzalez, M.-A. Gilles-Gonzalez, and M. Alam. 2009. Globins Synthesize the Second Messenger Bis-(3′–5′)-Cyclic Diguanosine Monophosphate in Bacteria. *J. Mol. Biol.* 388: 262-270.
